# A semantic segmentation scheme for night driving improved by irregular convolution

**DOI:** 10.3389/fnbot.2023.1189033

**Published:** 2023-06-12

**Authors:** Yang Xuantao, Han Junying, Liu Chenzhong

**Affiliations:** College of Information Sciences and Technology, Gansu Agricultural University, Lanzhou, China

**Keywords:** semantic segmentation, insufficient light, motion blur, generative model, irregular convolution

## Abstract

In order to solve the poor performance of real-time semantic segmentation of night road conditions in video images due to insufficient light and motion blur, this study proposes a scheme: a fuzzy information complementation strategy based on generative models and a network that fuses different intermediate layer outputs to complement spatial semantics which also embeds irregular convolutional attention modules for fine extraction of motion target boundaries. First, DeblurGan is used to generate information to fix the lost semantics in the original image; then, the outputs of different intermediate layers are taken out, assigned different weight scaling factors, and fused; finally, the irregular convolutional attention with the best effect is selected. The scheme achieves Global Accuracy of 89.1% Mean and IOU 94.2% on the night driving dataset of this experiment, which exceeds the best performance of DeepLabv3 by 1.3 and 7.2%, and achieves an Accuracy of 83.0% on the small volume label (Moveable). The experimental results demonstrate that the solution can effectively cope with various problems faced by night driving and enhance the model's perception. It also provides a technical reference for the semantic segmentation problem of vehicles driving in the nighttime environment.

## Introduction

In the field of intelligent transportation, the key to reducing traffic congestion and improving traffic efficiency lies in the optimization of vehicle autopilot algorithms (Wang et al., 2022; Ma et al., 2023). Semantic segmentation, as a pixel-level classification algorithm in the field of computer vision, is the basic algorithm for vehicles used to perceive external road conditions, which can help vehicles understand the semantics of people, vehicles, and obstacles precisely and quickly, and provide guidance for obstacle avoidance and path planning. The study of semantic segmentation is of great significance to reduce travel costs and improve vehicle utilization.

The current semantic segmentation models can be divided into two categories, one of which is based on convolutional neural networks such as FCN, DeepLab, U-Net, SegNet, and PSPNet (Chen et al., 2015, 2017a,b; Long et al., 2015; Ronneberger et al., 2015; Zhao et al., 2017; de Oliveira Junior et al., 2018). These networks perform feature extraction through convolution calculation, with improvement in organization and optimization structure. For example, Li et al. used MobileNetV2 as the backbone network to streamline the upsampling process, reduce the number of network parameters, and introduce a double attention mechanism to improve accuracy (Li and Wu, 2022). Zhong et al. proposed Faster-UNet, a lightweight semantic segmentation model based on UNet, which inherits the structural features of UNet encoding-decoding and combines multi-layer feature sensing capabilities to improve accuracy and make the model more lightweight on the Camvid dataset of road scenes (Zhong et al., 2022). Another class is based on Transformer architecture, such as VisionTransformer, SwinTransformer, and segfomer (Dosovitskiy et al., 2020; Liu et al., 2021; Xie et al., 2021); these models benefit from the superiority of the self-attentive mechanism and tend to achieve significant results in large data, high-calculus environments. For example, Jiang et al. (2023) used the Swin Transformer based on the self-attentive mechanism as the backbone network to extract microseismic first-arrival features, which can achieve high seismic feature pickup accuracy in low signal-to-noise ratio environments. Tian et al. (2023) merged features from the SegFormer encoder output at multiple scales in a cascade fashion while recalibrating the mapping of connected features based on channel contextual relationships, achieving excellent results on the UAVid and ISPRS Potsdam datasets. However, the above data are based on the annotation of normal semantic segmentation environments. The environment faced by cars during nighttime autonomous driving is more complex, involving factors such as dimness, defocus from high-speed driving, and motion blur. Ordinary convolution and transformer networks do not adapt well to these factors. For example, dilated convolution for semantic acquisition discontinuity causes information loss in dark areas, the transformer self-attention mechanism cannot achieve both high efficiency and low graphics memory simultaneously.

For motion blur, especially when dealing with dark-lighting blur in images, the common solutions are mainly divided into traditional algorithm-based solutions and deep learning-based solutions. The latter has strong generalization ability, uncomplicated tuning parameters, and other characteristics. Compared to the former it is more widely used in image deblurring research, such as when Zhang et al. (2022) proposed to use a deep self-coding network to encode the blur operator in the data set, and the encoded blurred operators to approximate the unknown blurred operators, which significantly improved the clarity of infrared images. Qing et al. (2022) proposed an image restoration algorithm based on wavelet domain ADMM depth network, and the accuracy exceeded the original algorithm on Set10, BSD68, and Urban100 datasets, and significantly reduced the operational complexity. However, all the above related research are aimed at the deblurring task itself, without introducing deep learning deblurring models for image recognition, detection, segmentation, and other tasks to preprocess the degradation of the dataset from noise interference.

To solve the above problem, this paper proposes a scheme. First, the generative model DeblurGAN (Kupyn et al., 2020) is used to deblur the blurred images; then, different weight scale factors are set up to fuse the intermediate layer outputs according to the heat map out of different intermediate layer features; finally, various attention mechanism modules are used in the main segmentation model for comparison experiments. The scheme has been significantly improved in accuracy and average IOU, and this idea of introducing the generative model into the semantic segmentation task can provide technical reference for other image noise reduction reanalysis tasks, especially for night driving type image segmentation problems.

## Experimental data analysis

**Figure 1:**
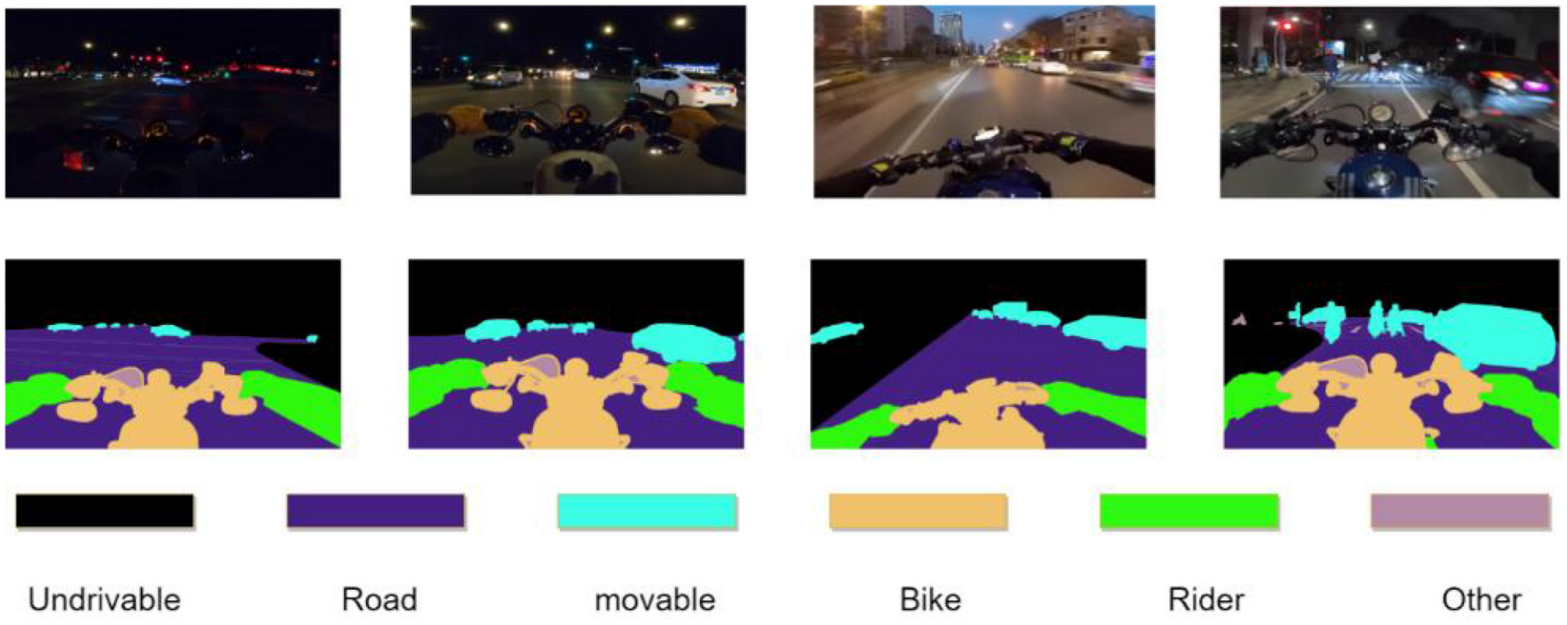
Data annotation style.

The data used in this paper come from the ACME_AI website. The images in this dataset were collected from a YouTube video of a motorcycle rider riding along a city street at night in Tucson, southern Pima County, Arizona, USA (latitude 32°13′_N longitude 110°58′_W). Each image size is 1,920^*^1,080, with the semantic segmentation annotation performed. In this paper, we divide the training set and test set into 8:2, with 160 images in the training set and 40 images in the test set, and process the original data to classify the labels into six categories: sky and neighborhood buildings (Undrivable), roads (Road), pedestrians and other vehicles (Movable), the rider's motorcycle (Bike), rider (Rider), and other (Other). As shown in Figure 1 below, the other(Other) class is the area that is not easily discerned by the model, or the semantic information of such an area is no longer discernible due to environmental interference, such as being too far, too dark, or having excessive blur. The loss of this class is not calculated in the model back propagation process.

**Figure 2:**
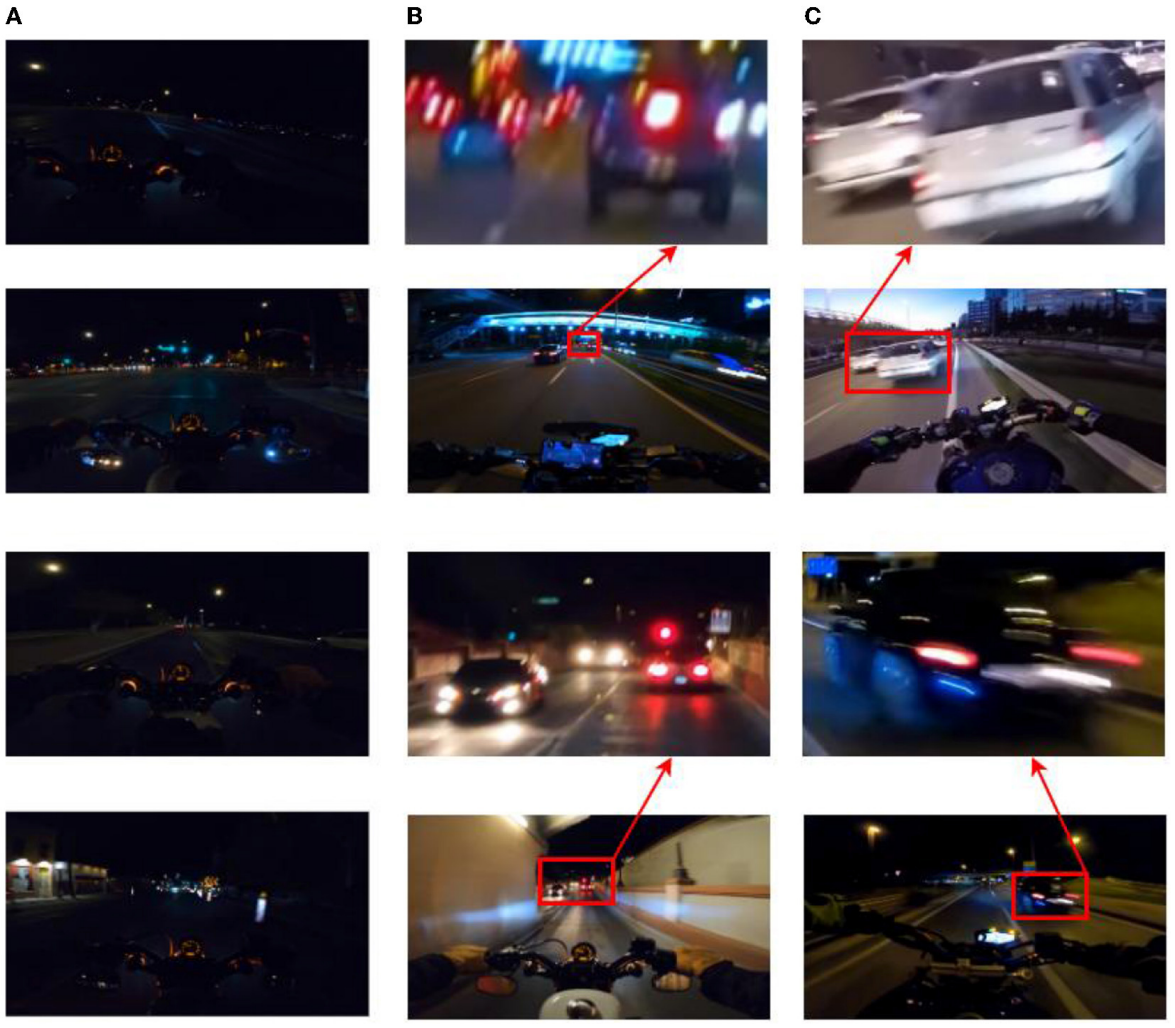
**(A)** Dark. **(B)** Out of focus. **(C)** Motion blur.

Due to the shooting process at night and because of the motorcycle's speed, the images face many problems both in the data labeling and experimental analysis stages, such as insufficient light, motion blur, and being out of focus. Some of the objects have been difficult to label accurately at the pixel level, and the supply of training samples is poor, with only 160 images, which makes the segmentation scheme more difficult to design as is shown in Figure 2.

## Experimental design

### Data pre-processing

#### Traditional algorithm for deblurring

Image blurring is a common visual degradation phenomenon in daily life, and the importance of deblurring is obvious in order to improve the accuracy of related image processing tasks. The deblurring algorithms can be divided into traditional algorithms and deep learning-based algorithms (Li and Shao, 2020). The process of traditional algorithms is mostly based on the estimation of the blur kernel, and the deconvolution operation is performed to find the approximate solution of the original image, which is simply described by a mathematical expression as follows (Equation 1).


(1) $$\begin{array}{l} p^{\prime }=k^{*}p+n \end{array}$$

*p*′ is the final obtained blurred image, *p* is the original image, *k* is the blur kernel between the imaging device and the target object due to interference factors such as dithering, and n is the additive noise in the environment.

The process of image deblurring is the process of inverse solving for *p* according to the information of *p*′. However, due to the uncertainty of *k*, the restoration algorithm is complicated, and it is difficult to repair pixel points whose values are too different from their surroundings. In this regard, this paper uses a depth generation model for image deblurring and achieves better clarity on in-motion, out-of-focus blurred night driving images.

#### Deep learning model for deblurring

**Figure 3:**
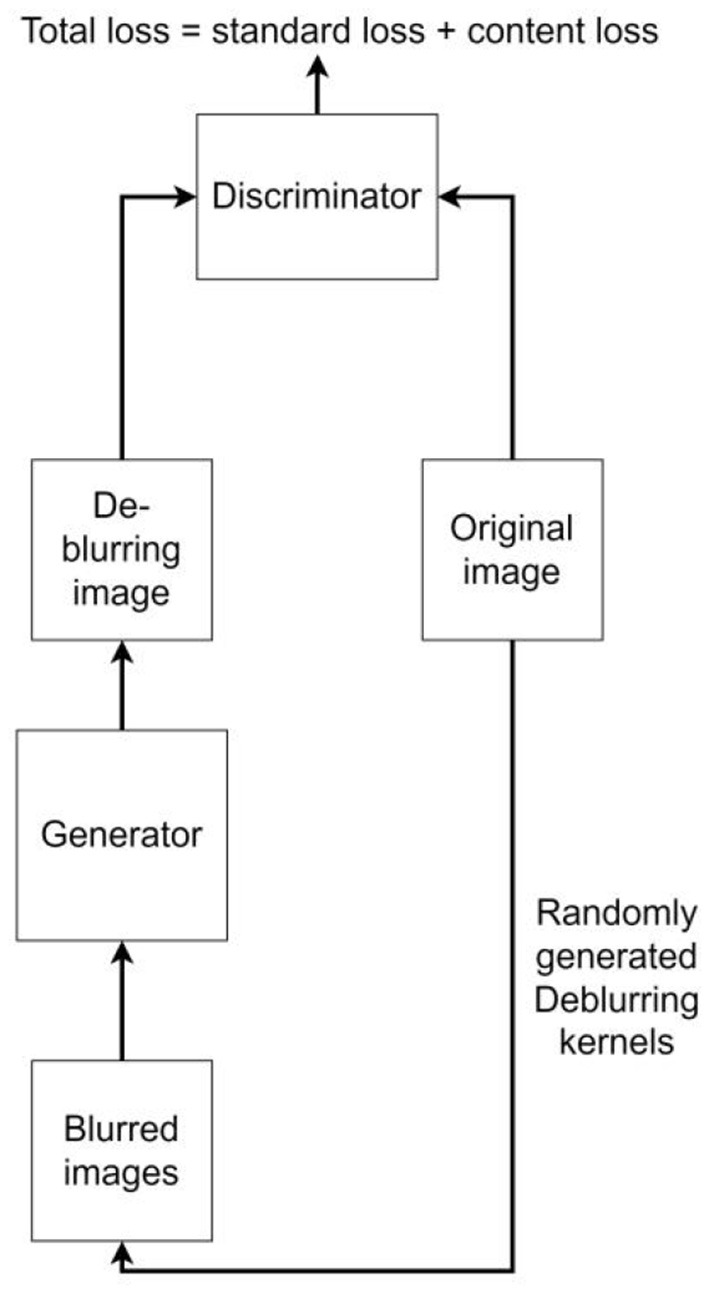
DeblurGAN algorithm (simplified).

**Figure 4:**
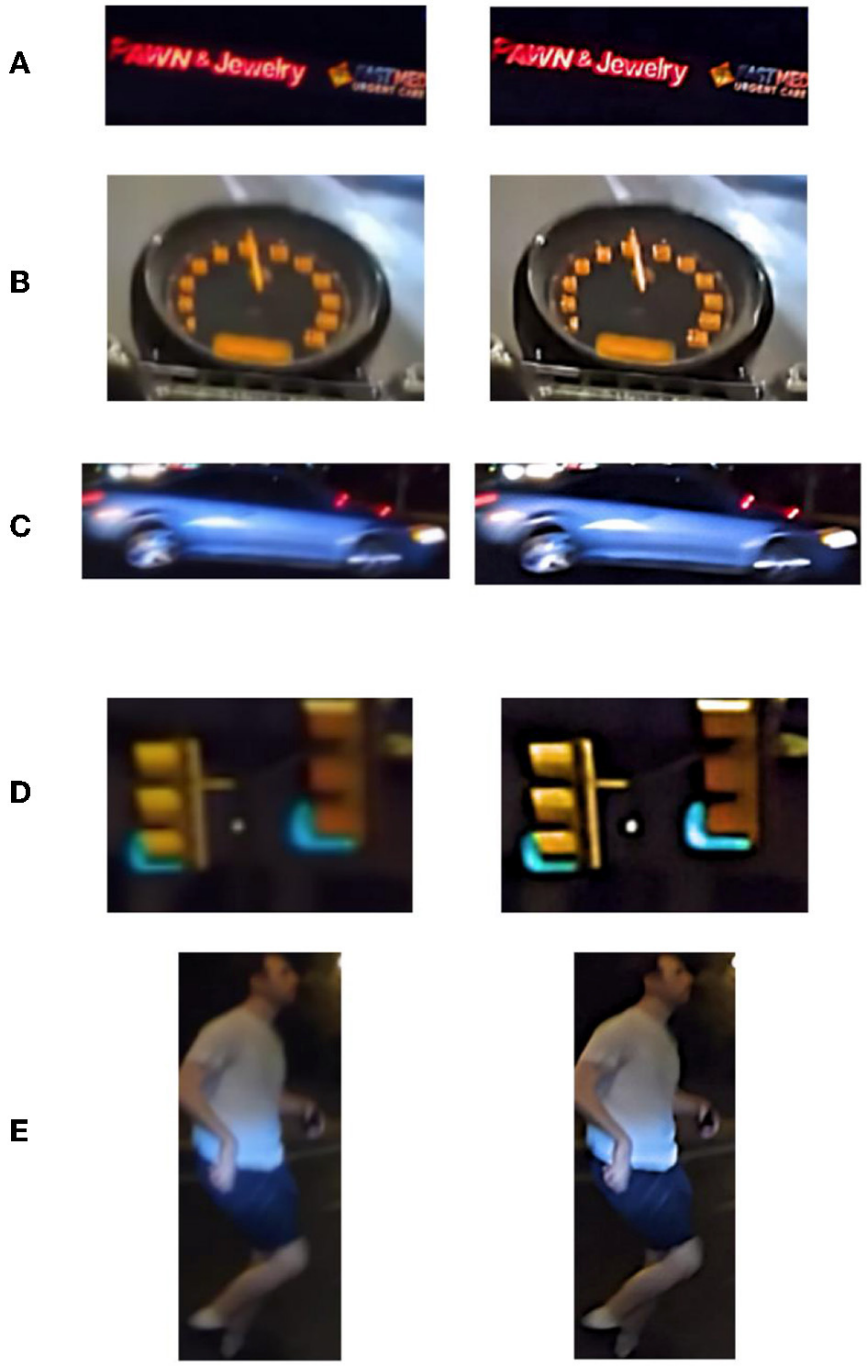
The DeblurGAN's effect, Left is the original image, right is the image after the model's processing. **(A)** Street sign. **(B)** Dashboard. **(C)** Vehicle. **(D)** Signal. **(E)** Pedestrian.

DeblurGAN was proposed by Isola et al. at CVPR2017, and the core idea is to treat deblurring as a special case of the image-to-image translation problem, where a clear image is blurred and then fed to a generator, and the output of the generator is computed as a loss with the original image. Compared with other deblurring networks [e.g., WGAN (Arjovsky et al., 2017) and WGAN-GP (Gulrajani et al., 2017)] DeblurGAN improves the loss function to be the sum of Content loss and standard loss Adversarial loss, which makes the model training more robust. The algorithm flow is shown in Figure 3. In this paper, we use DeblurGAN to deblur the 160 images in the training set, and Figure 4 shows the comparison of objects in some images before and after DeblurGAN processing (including Street signs, the dashboard, vehicles, signals, and pedestrians). Through observation, the images that are blurred due to nighttime defocus and vehicle driving motion are relatively clear after DeblurGAN processing.

### Benchmark model

**Figure 5:**
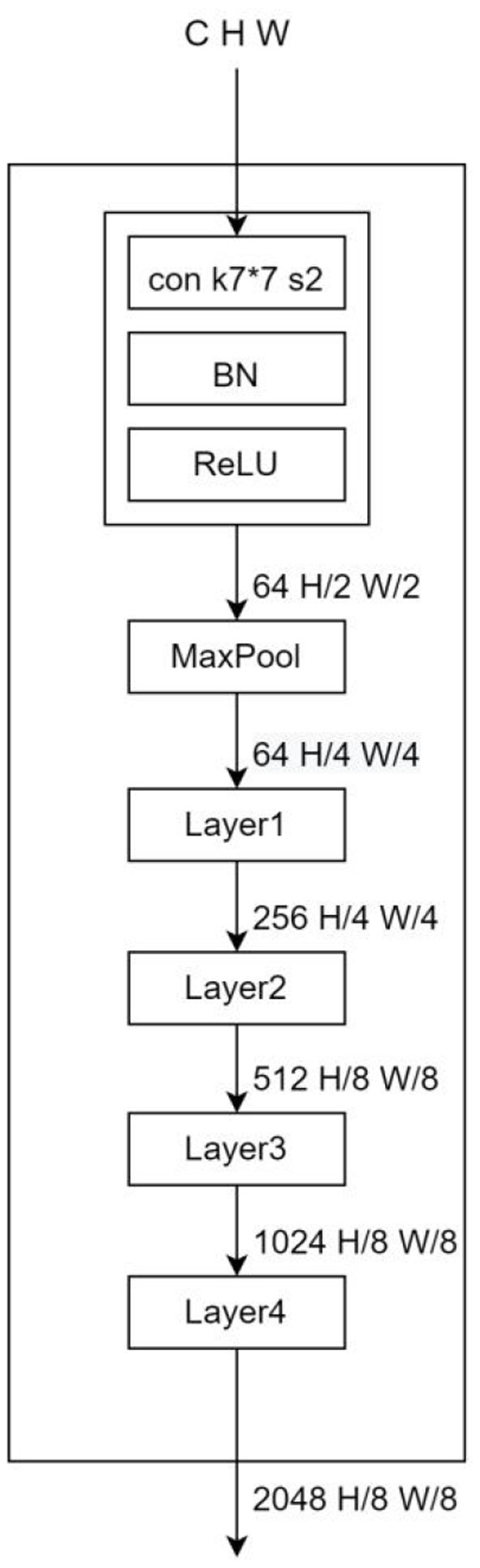
BackBone where k is the convolutional kernel size, s is the convolutional step size, and the hole convolution is added in Layers 3 and 4.

**Figure 6:**
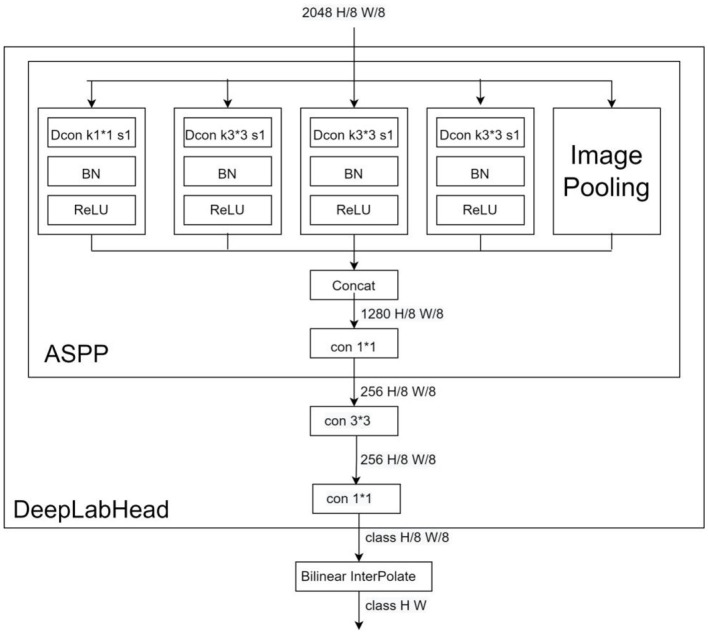
DeeplabHead Dcon for null convolution, Bilinear InterPolate for bilinear interpolation upsampling.

DeepLab series has become a very popular model in the segmentation field in recent years, and has demonstrated excellent performance in many datasets. In Deeplabv1, the dilated convolution is proposed for the first time instead of upsampling and downsampling to increase the receptive field of the model while keeping the feature scale unchanged, and CRF (conditional random field) is introduced to improve the ability of the model to capture local structural information. The prediction result of v1 has better edge details compared with older models. DeepLabv2 adds an ASPP (Atrous spatial pyramid pooling) module on top of v1, which is composed of multiple dilated convolutions with different expansion factors in parallel, aiming at stable segmentation of the target at a multi-scale level to obtain more semantic features of the image. DeepLabv3, the benchmark model in this paper, adds a BN (Batch Normalization) layer to the ASPP module and adds an ImagePooling to further enhance the contextual semantic extraction ability of the model, and then replaces the ASPP size 3^*^3 null convolution with a normal 1^*^1 convolution to preserve the effective weights in the middle part of the filter (It is found experimentally that the effective weight of the convolution kernel decreases as the expansion rate increases.). Meanwhile, the backbone network is replaced with ResNet, which deepens the number of dilated convolutions, and removes the CRF to streamline the output part of the model. The model structure is shown in Figure 5 (Network backbone) and Figure 6 (DeepLabHead with ASPP output part).

### Model improvement

#### Spatial semantic information for night driving

**Figure 7:**
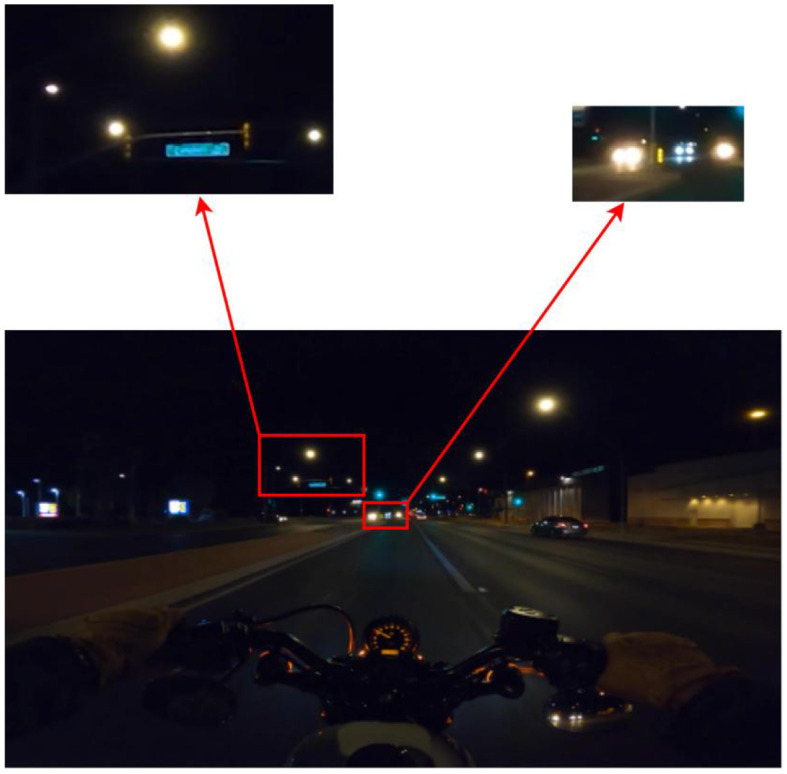
Different objects with high similarities at night.

The data in this paper is special; compared with the general semantic segmentation dataset, dark light, motion blur, defocus, and other issues make the model more sensitive to spatial information, shown in Figure 7. Here, the vehicle lights and street lights in the dim environment are both strong light sources, so in the process of fast driving and shooting the halo, it is difficult for the model to learn enough information from shape and boundaries alone to distinguish such labels. At this time, spatial semantics is an important feature (the light source close to the road and far from the sky is more likely to be a vehicle light source, while the opposite is more likely to be a building light source). Similarly, other objects with blurred textures and distorted structures during photography are also susceptible to spatial semantics.

The main reason why DeepLabv3 can achieve a better performance on the dataset of this paper (see the Experimental data analysis section of this paper) is its ASPP module, which uses the parallel structure of the dilated convolution with multiple expansion factors to fully integrate the multi-scale spatial semantics in the dark light environment. However, the dilated convolution itself has drawbacks in spatial semantic extraction, so the subsequent part of the experiment will compare the performance of the model with the grad-cam heat map between various modified models and reconstruct the model based on the effect of spatial semantics in dim light.

#### Disadvantages of the benchmark model

In DeepLabv3 backbone network, the neighboring pixels in the output feature map of the dilated convolution are obtained from the convolution of mutually independent subsets, which lack dependence on each other, while the spatial semantic information is a feature with strong dependence, and the data convolved by the dilated convolution will lose much spatial semantics. The multi-scale fusion feature of the ASPP module in the output head part (DeeplabHead), which can compensate for the semantic loss caused by the discrete extraction of features from a large number of dilated convolutions in the backbone network. This alone is not enough, however, so the extraction capability of the spatial semantics of the benchmark model in dark light needs to be improved to enhance the semantic segmentation accuracy for night driving.

#### Network analysis and restructuring

**Figure 8:**
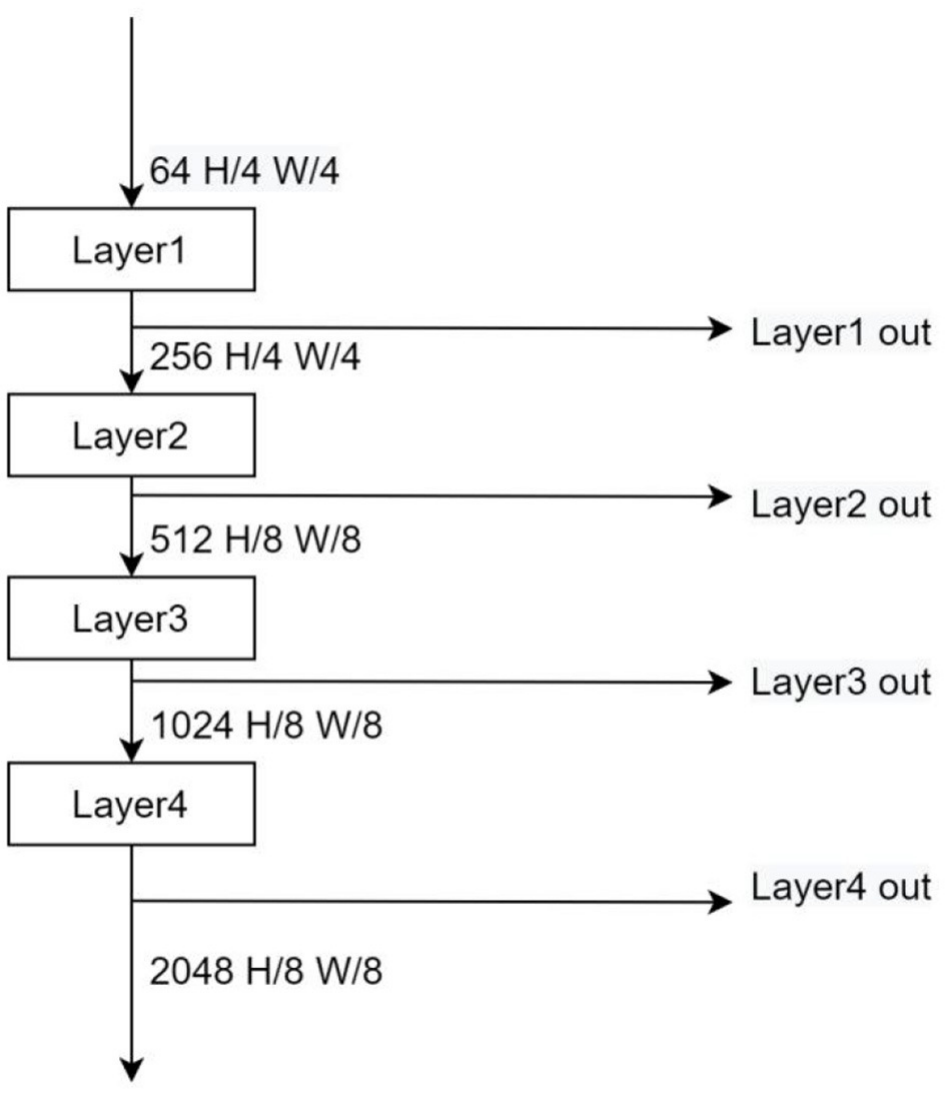
Extraction of the feature map of the intermediate layer output.

**Figure 9:**
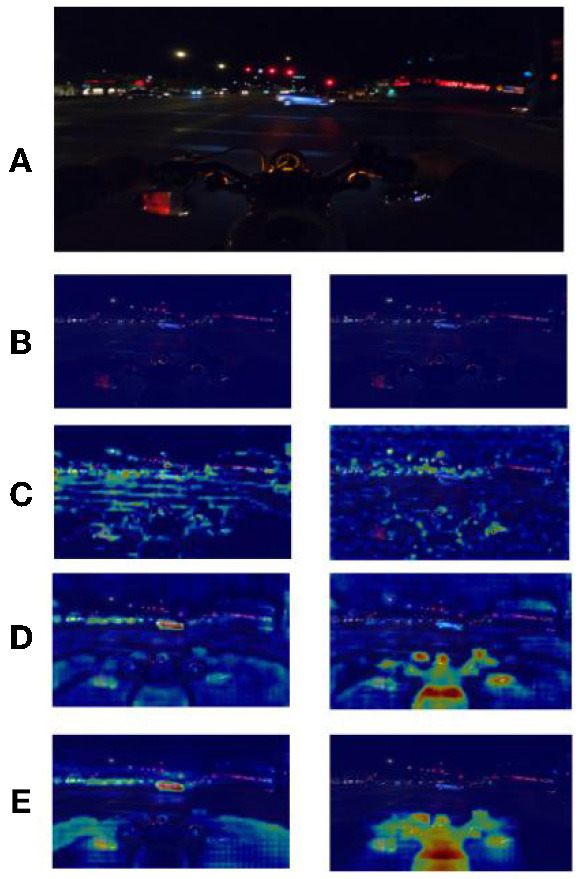
**(A)** is the input image, **(B–E)** are heat maps of Layers 1 to 4 (movable) on the left and (bike) on the right. Red is the area that the model focuses on.

In order to solve the problem of spatial semantic loss of the above benchmark model under dark light conditions, this experiment takes out the feature maps of the middle layer and fuses them with the original output after a series of downsampling processes. The Grad-CAM algorithm is used to analyze the attention regions of each layer. Grad-CAM (Gradient-weighted Class Activation Mapping) is an improved algorithm of CAM (Class Activation Mapping) (Selvaraju et al., 2017), which can help us analyze the attention regions of the network for a certain class. In this paper, we take out the feature maps of different stages in the backbone network (Figure 8), and plot their grad-cam heat maps for the categories Movable and Bike (Figure 9).

**Figure 10:**
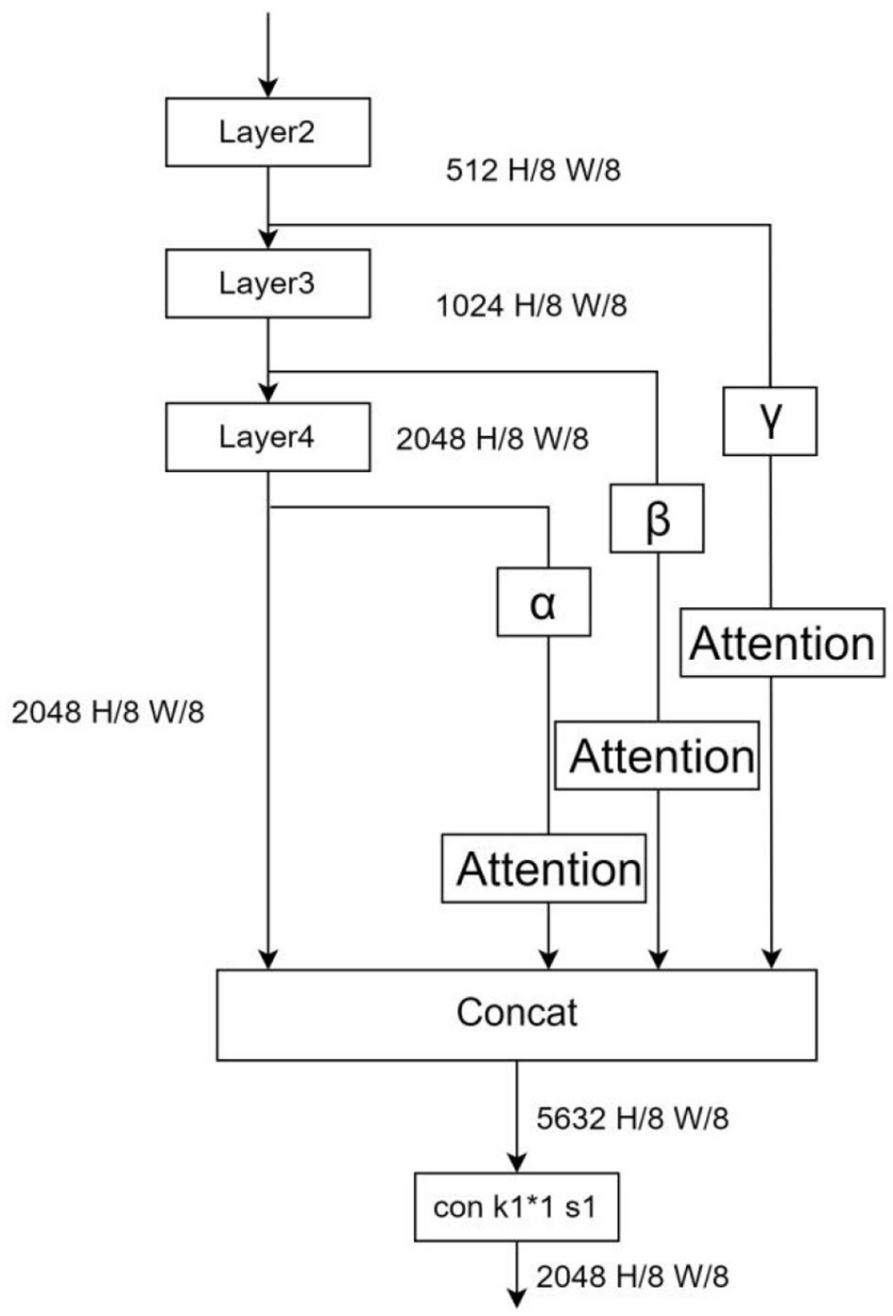
Improved model structure.

From Figure 9, it is easy to see that, as the network becomes deeper, the model focuses more and more on the right region of attention, and the deeper attention region is important more than the shallow one, the output feature map of Layer 1 even has no obvious focus region because of its shallow location. Based on the above observations, the outputs of networks Layers 2–4 (Layer 1 output has no obvious attention part and the spatial semantic contribution in these parts to dark light can be ignored, so the output of Layer 1 is discarded) are downsampled and spliced with the output of the original backbone network, and the features are passed through different attention modules with weight scaling factors (each pixel data of the output feature map is multiplied by fixed scale factor), and finally input to the ASPP module by 1^*^1 convolutional dimensionality reduction. See Figure 10.

#### Comparison of attentional mechanisms

**Figure 11:**
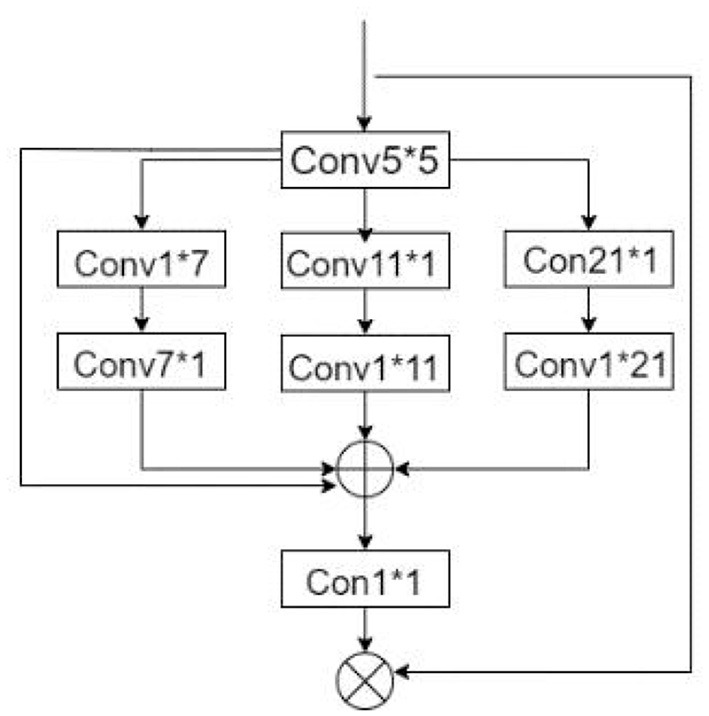
Irregular convolutional attention module.

The attention mechanism is increasingly used in deep learning tasks because of its excellent effect and plug-and-play convenience, but different attention modules have different performance capabilities for various work and data sets, such as SE attention mechanism (Squeeze-and-Excitation) using compression and reduction to get the weights of different data channels, so as to focus on the channels that contribute more to the current task. For spatially insensitive problems, such as image classification, the use of simple SE modules can yield excellent results (Hu et al., 2018); cbam (Convolutional Block Attention Module), an attention mechanism that combines two dimensions of channel attention and position attention, makes the model more sophisticated for spatial awareness problems, but the complexity of the module is higher than that of SE attention (Woo et al., 2018). The ca (Coordinate attention) attention mechanism captures the pixel dependence in *x* and *y* directions by means of coordinate information, which is a good trade-off between model accuracy and parametric overhead (Hou et al., 2021). In this paper, we compare the performance of various attention modules after embedding them into the network, and select the best irregular convolutional attention module. The module structure is shown in Figure 11, and the formula is shown in Equation (2).

Irregular convolutional attention (also called multiscale convolutional attention and later referred to as MSCA) was proposed by Guo et al. (2022). The module shown in Figure 11 consists of three parts: (1) a 5^*^5 convolution for aggregating local information, (2) multiple irregular convolutional pathways to capture contextual information, and (3) a 1^*^1 convolution to model inter-channel relationships.


(2) $$\begin{array}{l} Attention=Conv1^{*}1(\sum_{i=0}^{3}Scale_{i}(Conv5^{*}5(input)))\\ \;out=Attention^{*}input\\ \;Scale_{i}\;represents\;the\;i\text{-}th\;convolutional\;path \end{array}$$

#### Advantages of irregular attention on this dataset

**Figure 12:**
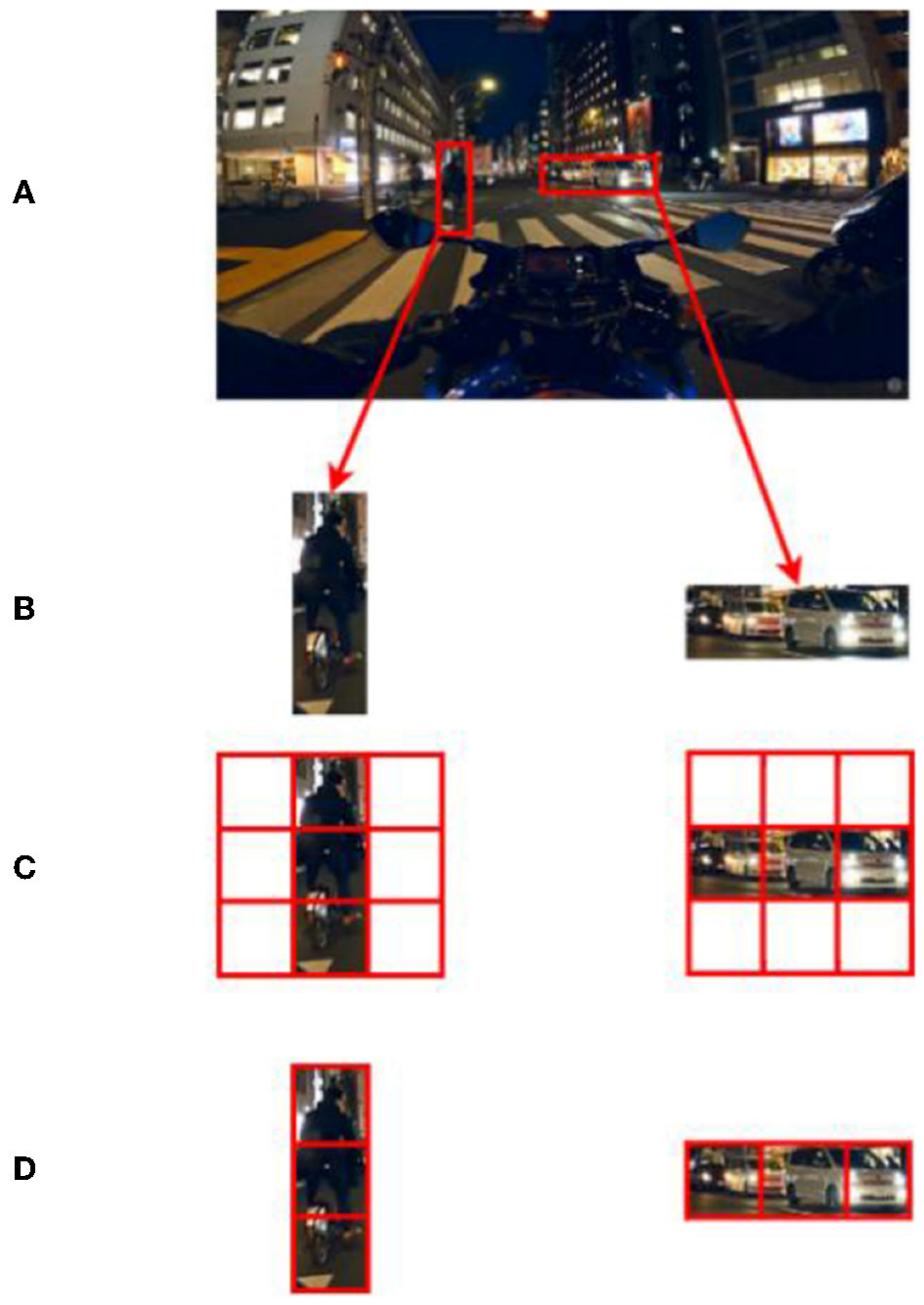
Irregular vs. regular convolution (for ease of observation, the size of the regular convolution kernel is simplified to 3*3 and the size of the irregular convolution kernel is simplified to 3*1 vs. 1*3). **(A)** Original image **(B)** motorcycle and car in the image **(C)** regular convolution **(D)** irregular convolution.

The entire MSCA module consists of a large number of irregular convolution layers. The irregular convolution of strips facilitates the fitting and segmentation of objects with irregular shapes (most of the objects to be segmented in this experiment are not standard squares, such as vehicles and pedestrians, see Figure 12). Irregular convolution, on the other hand, extracts information in a region that fits better with the target shape and maximizes the use of the parameters of the convolution kernel (Figure 12D) to extract less redundant information that interferes with the object, thus improving the judgment of the object boundary. In images where the underlying semantics (texture and edges) are corrupted by dark light, blur, etc., the convolution kernel accurately extracts the semantic information of the object and facilitates the fine fitting of label boundaries for each category.

## Analysis of experimental results

### Experimental environment

The experimental machine configuration and some of the hyperparameters are as follows shown as Tables 1, 2.

**Table 1:** Experimental machine configuration.

**Device/environment**	**Version/capacity**
Processor	Intel(R) Core(TM) i7-11800H @ 2.30 GHz
GPU	Nvidia GeForce RTX 3060 6 g
Framework	Python 3.7.0 Pytorch1.12 cuda11.3
Operating system	Windows 11
Memory	16 g

**Table 2:** Hyperparameter settings.

**Parameters**	**Value/method**
Epoch	50
BatchSize (train)	4
BatchSize (test)	1
Initial learning rate	0.0001
Regularization	L2 Regularization

### Learning rate update strategy and training curve

**Figure 13:**
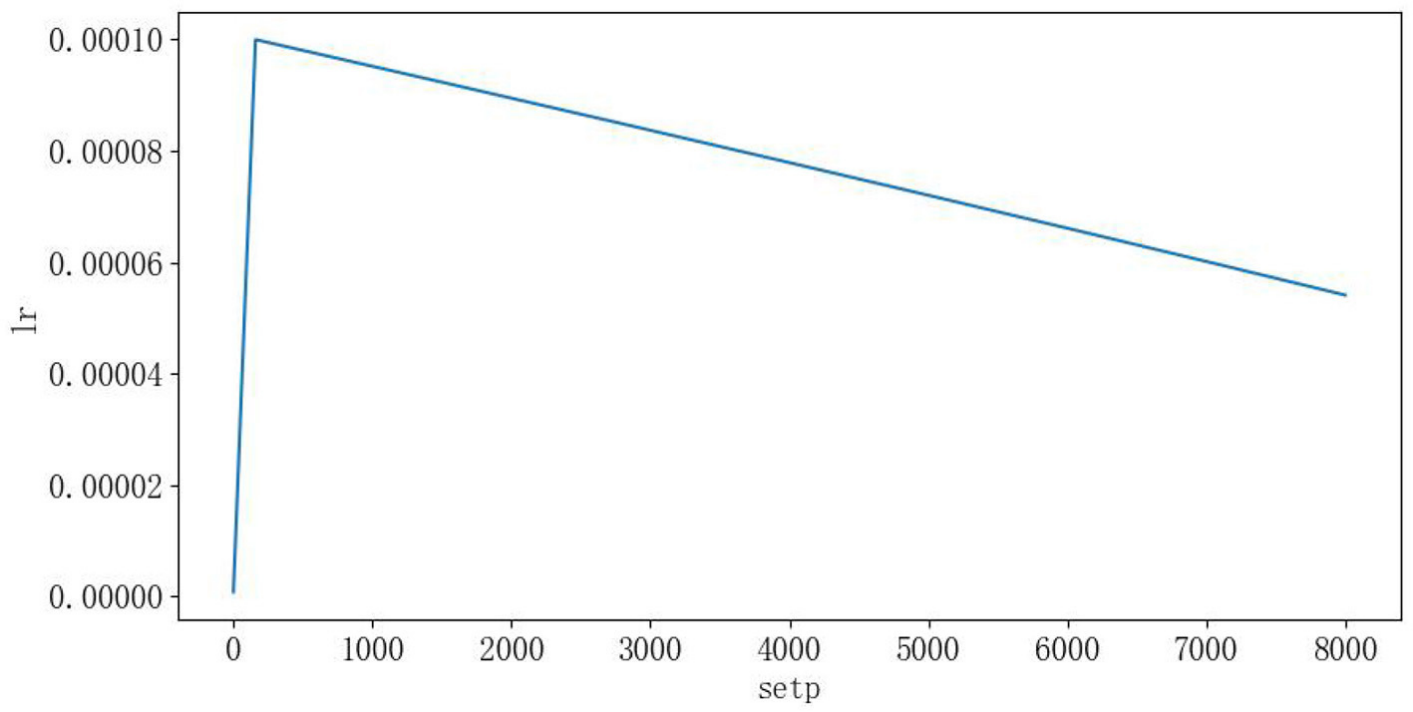
Learning rate curve (this experiment has 160 steps per Epoch and the horizontal axis is 8,000 long).

**Figure 14:**
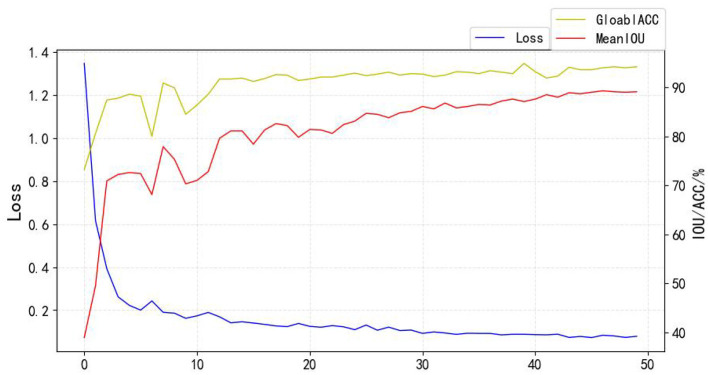
Training process.

The experiments are based on the ploy learning strategy proposed in DeepLabv2, and the following two improvements are made: (1) the ploy learning adjustment strategy in the original paper is based on epoch, while in this paper, when training the model, the backbone network is loaded with the pre-training weights of DeepLabv3 on the COCO dataset and the weights of the added modules are initialized using a normal distribution (Lin et al., 2014). Therefore, the adjustment strategy in this paper is subdivided into individual steps in each epoch, and the fine dynamic adjustment of the learning rate is conducive to the stable decrease of the model loss; (2) by using the Warmup strategy, the model is insensitive to the disturbance of random weights under the small learning rate of warm-up, and so the pre-set learning rate can be selected after the relative stability, which makes the model converge faster and better. The Ploy adjustment formula is shown in Equation (3), the learning rate curve is shown in Figure 13, and the training curve (Loss, MeanIOU, GlobalAccuracy) is shown in Figure 14:


(3) $$\begin{array}{l} 1-\frac{\left(x-WarmEpoch^{*}Step\right)}{{\left({\left(Epoch-WarmEpoch\right)}^{*}Step\right)}^{0.9}} \end{array}$$

*x* is the current number of steps, WarmEpoch is the number of epochs for training warm-up, and Step is the total number of steps in an Epoch.

### Evaluation indicators

The evaluation metrics of this experiment are Global Accuracy and Mean IOU for all classes (higher Global Accuracy means that the model recognizes a certain type of pixel point better, and a higher Mean IOU means that the model has a better overall segmentation for a certain type of pixel.), shown in Equation (4)


(4) $$\begin{array}{l} Global\;Accuarcy:\frac{\sum_{i=0}^{n}p_{ii}}{\sum_{i=0}^{n}(p_{ii}+\sum_{j=0}^{n}p_{ij})}\;\\ Mean\;IOU:\;\frac{\sum_{i=0}^{n}p_{ii}}{\sum_{i=0}^{n}(\sum_{j=0}^{n}pij+\sum_{j=0}^{n}pji-p_{ii})} \end{array}$$

*n* is the *n* segmented entities for this task, *p*_*ij*_ is the number of pixels of class *i* objects identified as class *j*, and *i* is not equal to *j* in Global Accuracy.

### Performance of each configuration

As seen from the data in Table 3, the scale factors α, β, *and γ* decreasing in order is better, with 0.7, 0.5, 0.3 ratio being the best, which further proves the characteristic of the middle layer heat maps as analyzing above. The region of interest of the deep layer features is more accurate than the shallow layer, and the weight proportion of the deep layer feature map should be higher than the shallow layer when fusing multiple feature maps; as seen from the data in Table 4, MSCA attention is better than other control modules on this dataset, mainly because of the adaptation of the MSCA irregular convolutional pathway for irregular object labels, and the design of MSCA is more intuitive and targeted on the night driving dataset compared to other means of solving fine segmentation of edges.

**Table 3:** Performance of proportional factor combinations.

**α**, **β**, **γ Combination**	**MeanIOU**	**GlobalAccuracy**
0.9, 0.7, 0.5	+1.9%	+1.3%
0.7, 0.5, 0.3	+2.5%	+1.2%
0.5, 0.5, 0.5	+0.7%	+1.2%
0.3, 0.5, 0.7	−0.5%	+0.9%
0.5, 0.7, 0.9	−1.0%	+1.4%

**Table 4:** Various attentional performance.

**Attention**	**MeanIOU**	**GlobalAccuracy**
SE	+2.0%	+1.0%
Cbam	+2.4%	−3%
Coordinate Attention	+0.3%	−1.4%
MSCA	+4.7%	+0.8%

### Model comparison

The following semantic segmentation models are compared with this paper's model (Tables 5, 6) (the improved model in this paper is named MA-LAB-res101). The global segmentation performance (Global Accuracy and Mean IOU) and the small volume object segmentation performance (Accuracy and IOU of both small volume labels Moveable and Rider) are analyzed from two perspectives. The effectiveness of this paper is analyzed from two perspectives.

**Table 5:** Accuracy/%(× indicates that the model takes up too much video memory, resulting in overflow).

**Models**	**Architecture**	**Undrivable**	**Road**	**Moveable**	**Bike**	**Rider**	**Global**
FCN-8s-ResNet50	Convolution	90.8	91.3	42.6	94.7	53.0	85.3
FCN-8s-ResNet101	Convolution	92.4	93.7	52.0	94.2	80.8	89.2
Lraspp-MobileNetv3	Convolution	89.1	86.3	53.6	91.0	18.5	80.7
DeepLabv3-ResNet50	Convolution	87.2	94.3	17.3	96.5	45.2	82.5
DeepLabv3-ResNet101	Convolution	94.4	93.4	78.1	95.7	90.4	92.9
Segfomer-b0	Transformer	89.9	87.9	51.0	95.1	46.9	84.3
Segfomer-b1	Transformer	91.6	87.8	47.0	94.5	49.1	84.7
Segfomer-b2	Transformer	92.6	91.3	58.0	97.1	50.7	87.4
Segfomer-b3	Transformer	×	×	×	×	×	×
Segfomer-b4	Transformer	×	×	×	×	×	×
Swin-T	Transformer	87.6	86.7	56.0	90.1	55.2	83.3
Swin-B	Transformer	90.1	87.5	59.7	93.1	67.0	89.2
Swin-L	Transformer	×	×	×	×	×	×
MA-LAB-res101	Convolution	96.8	91.8	83.0	97.7	86.0	94.2

**Table 6:** IOU/%.

**Models**	**Architecture**	**Undrivable**	**Road**	**Movable**	**Bike**	**Rider**	**Mean**
FCN-8s-ResNet50	Convolution	83.0	77.8	38.9	74.5	50.2	64.9
FCN-8s-ResNet101	Convolution	85.2	83.2	49.6	81.9	72.5	74.5
Lraspp-MobileNetv3	Convolution	81.2	74.1	37.8	65.9	17.5	55.3
DeepLabv3-ResNet50	Convolution	82.9	73.4	17.1	69.0	43.7	57.2
DeepLabv3-ResNet101	Convolution	90.5	85.5	60.1	90.4	82.7	81.9
Regfomer-b0	Transformer	83.6	76.7	39.5	73.0	42.5	63.1
Regfomer-b1	Transformer	84.8	78.0	37.3	72.6	43.8	63.3
Regfomer-b2	Transformer	87.4	82.3	49.5	76.4	46.3	68.4
Regfomer-b3	Transformer	×	×	×	×	×	×
Regfomer-b4	Transformer	×	×	×	×	×	×
Swin-T	Transformer	88.7	72.5	23.0	61.2	50.1	59.1
Swin-B	Transformer	89.1	74.0	33.2	80.1	52.3	65.7
Swin-L	Transformer	×	×	×	×	×	×
db-LAB-res101	Convolution	91.4	86.8	81.0	90.9	86.8	89.1

#### Global segmentation results

In order to verify the improvement of the global semantic segmentation performance of the improved scheme, we focused on the indicators Global Accuracy and Mean IOU. From the analysis of the above data, it is easy to see that the traditional convolutional segmentation models and the Transformer segmentation models are not very different in Global Accuracy, but the Mean IOU of the Transformer models is mostly concentrated in the low range of 60% to 70%. Meanwhile, although the traditional convolutional models can balance the hardware resources and global segmentation performance well, the best one, DeepLabv3-ResNet101, is still not excellent. The Global Accuracy and Mean IOU of the improved scheme proposed in this paper are 94.2 and 89.1%, which exceed the best performance of DeepLabv3- ResNet101 by 1.3 and 7.2%.

#### Small label segmentation results

**Figure 15:**
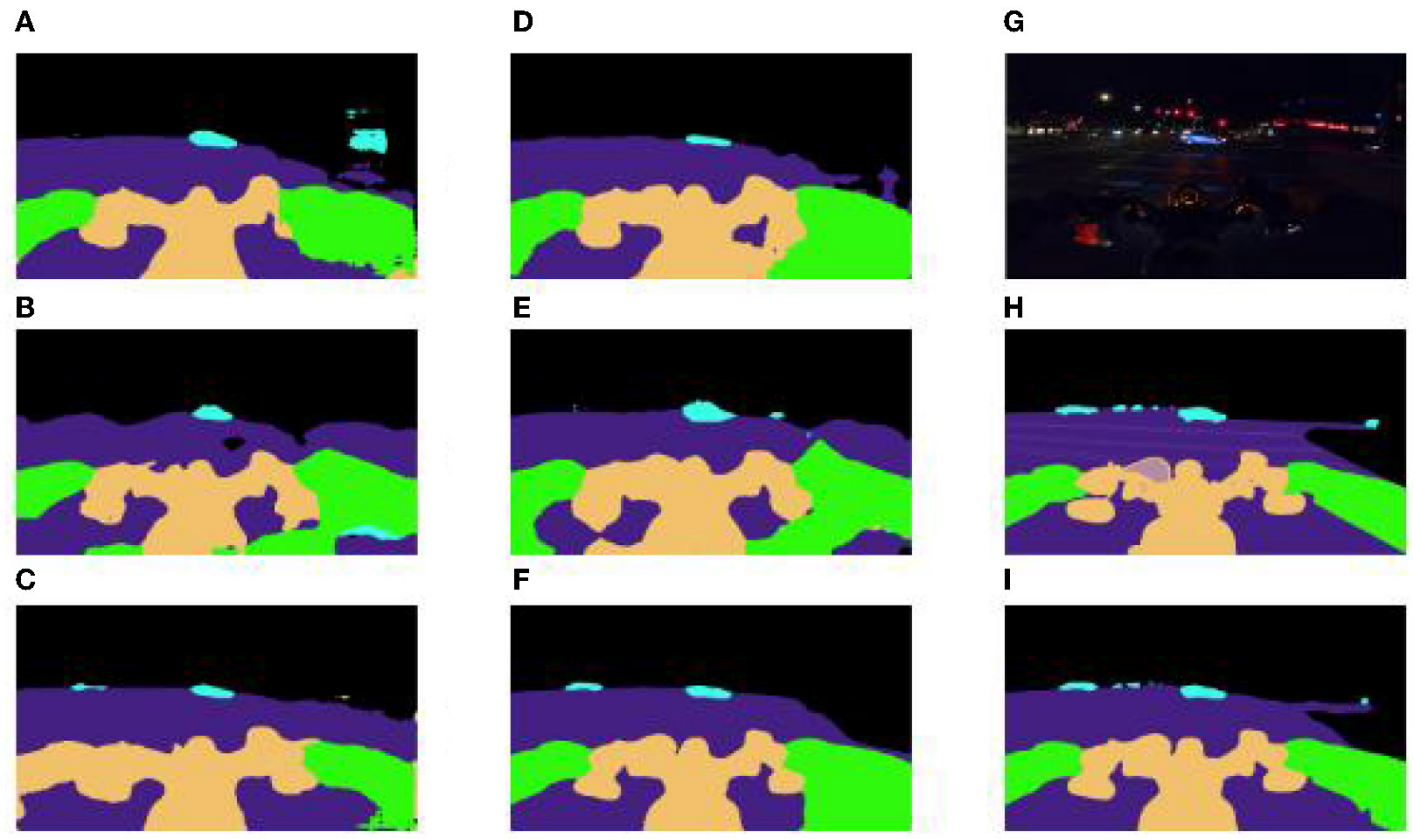
**(A)** FCN = 8s-ResNet50 **(B)** segformer-b0 **(C)** DeepLabv3-resnet150 **(D)** FCN = 8s-ResNet101 **(E)** segformer-b1 **(F)** DeepLabv3-resnet101 **(G)** real pictures **(H)** real labels **(I)** this paper scheme (db-LAB-res101).

In order to verify the adaptability of the improved scheme for dark light, motion blur, and other interference factors, we focus on analyzing the performance of small volume label segmentation which is susceptible to interference. For Undrivable, Road, and Bike, three large volume labels, each control model performs well, but the prediction of Rider and movable, two small volume labels, is not good. From Figure 15, we can see that, in part of the network, due to interference factors such as dark light and motion blur, the boundary between the rider and the motorcycle is not clear, and the network divides the rider and the motorcycle into a whole, while the vehicle, as the most difficult small target to be segmented in the night driving scene, is easily ignored by the model, and then directly segmented into road sections or the background. The segmentation scheme proposed in this paper complements the spatial semantics in dark light and overcomes the poor performance of small object segmentation in the night road environment, and achieves IOU of 81.0 and 86.8% in Movable and Rider categories, which exceed the best performance of DeepLabv3 in the control model by 20.9 and 4.1%.

## Conclusion

In this paper, the innovation for the segmentation scheme focuses on data pre-processing and model design. Firstly, using generative models for image deblur can provide stronger noise removal performance than traditional algorithms, and also avoids the tedious process of traditional image algorithms. Secondly, during the process of model design, the different combinations of fusion scale factors is chosen, and the network is reconstructed to complement the spatial semantics in dark light. Multiple attention modules are compared to demonstrate the superiority of irregular convolution in the extraction of boundary information for the night driving semantic segmentation task; finally the optimal model adapted to the night driving environment is found.

However, there are still many areas for improvement in this scheme. For example, although the model exceeds the benchmark model in some indicators, such as Accuracy and Mean IOU, there is a small increase in the number of model's parameters, and the light weight of the model is a problem that must be faced when implementing autonomous driving algorithms. In future research, a series of light weight techniques such as knowledge distillation, pruning, quantization, and weight sharing will be used to improve the structure of the db-LAB-res101 model proposed in this paper, so as to reduce the resource consumption of the model in engineering applications and improve the computing speed. We will also perform migration to different areas and will apply the theory of the solution proposed in this paper not only in semantic segmentation tasks, but will also transfer the model studied to more complex night driving segmentation tasks, such as example segmentation and panoramic segmentation, in order to verify the adaptability of this paper's solution to multiple types of night driving segmentation tasks. We will also further investigate the combination of weighting factors in the middle layer to obtain improved precision on the values, as well as to demonstrate that the resulting parameter combinations can be adapted to different sets of night-time driving data.

## Data availability statement

The original contributions presented in the study are included in the article/supplementary material, further inquiries can be directed to the corresponding author.

## Author contributions

YX developed the idea, wrote the main manuscript text, designed and performed the experiment, and checked the data. HJ and LC provided funding and devices and helped with article revision. All authors reviewed the manuscript. All authors contributed to the article and approved the submitted version.
